# Assessment of Physical Fitness Following a 12-Week Physical Exercise Program Among Adults Attending Wellness Centers at the Primary Health Care Corporation, Qatar: A Retrospective Study

**DOI:** 10.7759/cureus.81096

**Published:** 2025-03-24

**Authors:** Anees A Alyafei, Aysha H Hussein, Hind Daoud Abdel Haleem AlDaoud, Stephanie E Escarmoso, Sara Tariq Al Abdulla

**Affiliations:** 1 Wellness Programs, Preventive Health, Primary Health Care Corporation, Doha, QAT

**Keywords:** body mass index: bmi, fat mass, muscular endurance, physical exercise, physical fitness, vo₂ max, waist circumference

## Abstract

Background

Physical fitness (PF) is a critical determinant of health, influencing cardiorespiratory endurance, muscular strength, and overall metabolic function. In Qatar, sedentary lifestyles and physical inactivity are prevalent, contributing to increasing rates of obesity and non-communicable diseases. Structured physical exercise programs, integrated into community wellness centers, offer a practical intervention to improve PF, yet their real-world effectiveness remains under-evaluated in the local context.

Methods

This retrospective study analyzed data from 739 adults who completed a 12-week structured physical exercise program at seven wellness centers operated by Primary Health Care Corporation (PHCC), Qatar, between January 2022 and December 2023. The program consisted of three weekly supervised sessions combining aerobic and resistance exercises, with heart rate monitored to maintain moderate intensity (≥70% of maximum heart rate). Pre- and post-program assessments measured VO_2_ max (using the Cooper 12-minute test), muscular endurance (push-ups, wall sit, plank), and anthropometric data of weight, body mass index (BMI), waist circumference (WC), and fat mass. Paired t-tests assessed changes in the means before and after the physical exercise program, and correlation analysis explored relationships between fitness improvements and demographic or anthropometric factors.

Results

After 12 weeks, VO_2_ max increased significantly (mean difference = 2.47 mL/kg/min; p < 0.001), alongside improvements in muscular endurance (push-ups: +4 reps; wall sit: +18 seconds; plank: +16.77 seconds; all p < 0.001). Significant reductions were observed in weight (-0.95 kg), BMI (-0.27 kg/m²), WC (men: -0.99 cm; women: -2.34 cm), and fat mass (-1.42 kg) (p < 0.001). Correlation analysis revealed a weak negative correlation between age and VO_2_ max change (r = 0.061), indicating that younger participants tended to show more remarkable aerobic improvement. Higher BMI and fat mass also correlated negatively with VO_2_ max gains (r = -0.094 and r = -0.083, respectively). At the same time, gender showed minimal correlation with fitness changes, suggesting that baseline body composition and age influence exercise response more than sex.

Conclusion

The structured 12-week exercise program significantly improved PF among adults in community wellness settings. These findings support integrating exercise prescriptions into primary care, to enhance population-level fitness and prevent chronic disease in Qatar.

## Introduction

Physical fitness (PF) is a collection of attributes that influence an individual’s capacity to engage in physical activities. These attributes are typically divided into health-related and skill-related components. Some of PF's health-related components include cardiorespiratory and muscular endurance [1].

According to the American College of Sports Medicine, the volume of oxygen maximum (VO_2_ max), also known as aerobic fitness or cardiorespiratory endurance, is the peak rate at which an individual can utilize oxygen during exercise. This rate reflects the body’s efficiency in using oxygen to produce energy and is a primary measure of cardiorespiratory fitness. The VO_2_ max is usually expressed in milliliters of oxygen per kilogram of body weight per minute [2]. Conversely, muscle endurance is defined as the ability of a muscle or group of muscles to sustain repeated contractions or to continue applying force against a fixed resistance for an extended period, typically measured in seconds [3].

Age and gender significantly influence PF levels and their changes over time [4]. Physiological declines, such as reduced muscle mass, lower metabolic rates, and decreased cardiorespiratory efficiency, become apparent as individuals age. Furthermore, gender differences affect fitness changes, with males typically showing higher VO_2_ max values due to increased body mass. These variations highlight the importance of personalized fitness program interventions [5].

Anthropometric measurements, including body mass index (BMI), body fat mass, and muscle mass, are closely linked to changes in PF metrics. A higher body fat percentage is associated with lower VO_2_ max levels, whereas an increase in fat-free mass contributes to improved VO_2_ max in healthy young adults [6]. Additionally, a strong negative correlation exists between the total body fat percentage and muscle strength and endurance [7]. These connections emphasize the importance of maintaining optimal body composition to enhance PF outcomes.

Overall, evidence suggests that increasing the intensity of physical exercise enhances VO_2_ max through aerobic training, as first demonstrated by Hargens and later corroborated by Crowley et al. [8,9]. Buffart et al. [10] conducted a meta-analysis examining the impact of resistance exercise on muscle endurance in older adults within community-based settings. The findings demonstrated that resistance training protocols significantly enhanced muscular endurance, particularly those utilizing moderate to high repetition ranges. These improvements are clinically relevant, contributing to greater functional capacity, autonomy in daily activities, and overall quality of life [11].

Qatar’s Primary Health Care Corporation (PHCC), a governmental leading primary care provider for the community, has implemented wellness initiatives to boost community health by targeting significant prevalent risk factors, including physical inactivity. The PHCC has integrated wellness facilities within seven health centers, offering resources such as swimming pools, gyms, and relaxation spaces. These centers are linked with family medicine, healthy lifestyle, and health coaching clinics. The wellness services are an opportunity to evaluate the effect of structured physical exercises on different aspects of PF.

In this study, adult participants underwent a 12-week structured physical exercise program as part of the wellness program. Patients participated in pre- and post-assessments, including measuring VO_2_ max and muscular endurance, to assess the program's impact on key elements of PF. This study aims to quantify changes in participants' VO_2_ max throughout the 12-week intervention, as shown by the differences in pre- and post-assessment results. Furthermore, the study investigates how sociodemographic factors and anthropometric measurements relate to differences in VO_2_ max and muscular endurance among participants. The 12-week intervention period was informed by evidence from exercise physiology literature, which demonstrates that measurable improvements in PF commonly occur within 8-16 weeks of structured physical training. This duration also aligns with the standardized timelines used in institutional wellness and preventive health programs.

## Materials and methods

Study design and setting

Our analysis relied on information collected from the Electronic Medical Record of seven PHCC-affiliated wellness centers over the period from January 2022 to December 2023, allowing us to explore these facilities in depth.

Study population

Our study focused on adults aged 18 and above, including both men and women, who completed a 12-week structured exercise program at the seven PHCC-operated wellness centers.

Intervention

A 12-week exercise program at all of the wellness centers located within primary care facilities was implemented. This program was designed to deliver three weekly sessions, lasting 60 minutes each. During these sessions, participants engaged in moderate-intensity exercises that kept their heart rates above 70% of their maximum heart rate, monitored in real-time to ensure consistency.

Professional trainers standardized the exercises, which included aerobic activities, resistance training, and detailed warm-up, training, and cool-down phases.

To ensure consistency, all exercise sessions followed a structured 12-week protocol developed in alignment with PHCC guidelines. Each session was delivered by certified gym instructors trained under a unified orientation program. The duration, intensity, and progression of exercises were pre-defined across all PHCC wellness centers. Likewise, all pre- and post-intervention fitness assessments (VO_2_ max tests, endurance evaluations, and anthropometric measurements) were conducted using validated tools and standardized procedures. Instructors used the same instructions, timing devices, and field test protocols (e.g., Cooper 12-minute run and plank hold) to ensure uniformity across participants and centers.

The program was closely supervised, with attendance tracked through the medical records; participants needed to attend at least 85% of the sessions to qualify for the final analysis. This threshold was selected based on exercise adherence literature, where ≥85% participation is considered sufficient to elicit significant physiological benefits and ensure intervention effectiveness. To boost attendance, reminders were sent via SMS and live phone calls. All participants underwent pre- and post-program evaluations, including assessments of PF and anthropometric measurements. All participants provided written informed consent before enrollment in the physical exercise program and saved it in their electronic medical records.

Sampling strategy

A consecutive sampling approach was adopted, incorporating all adult patients who completed the program and provided two complete data sets. This ensured that every patient record meeting our criteria within the study timeframe was included, free from any predefined selection criteria.

Eligibility criteria

Inclusion Criteria

Adults aged 18 years and above who were enrolled in the PHCC Wellness Centers’ 12-week physical exercise program between 2022 and 2023 were eligible. Participants were required to be medically cleared for physical activity by PHCC healthcare professionals, not currently engaged in any structured physical training, and willing to participate in pre- and post-program assessments.

Exclusion Criteria

Participants were excluded if they had unstable cardiovascular disease, uncontrolled hypertension or diabetes, recent surgery or injury limiting physical activity, or pregnancy. Individuals who missed more than 15% of the supervised sessions were also excluded from the final analysis. The study excluded patients with incomplete or missed critical data, patients who were currently taking weight management medications, and patients who reported exercising in private gyms during the same study period.

Data collection

Data was collected from PHCC's electronic records system for patients who completed a 12-week structured physical exercise program and underwent PF assessments. Sociodemographic information, including age in completed years and gender, was extracted. Pre- and post-intervention data on VO_2_ max (measured in mL/kg/min) and muscular endurance, which included the number of push-ups performed until exhaustion, wall sit, and plank exercises (measured in seconds), were analyzed to assess improvements in fitness over the intervention period. Anthropometric measurements included weight in kg, waist circumference (WC) in cm, BMI in kg/m², and fat mass in kg. The deidentified research data was stored securely in a password-protected Microsoft Excel® file (Microsoft Corporation, Redmond, WA, USA), accessible only to authorized research team members.

Quality measures

The PHCC Institutional Review Board approved the study proposal, ensuring that all procedures adhered to PHCC’s ethical standards. Participant confidentiality was ensured by anonymizing and managing personal data according to institutional guidelines. Consistent quality control measures were implemented throughout the study to maintain data integrity.

A standardized, institutional review board-approved data extraction protocol was developed to identify and record data from the electronic record in a systematic manner.

The study members performed recurring reliability checks to verify the consistency of patient record reviews, with discrepancies resolved through consensus or additional review by the principal researcher. Only complete and comparable datasets were included in the final analysis, and regular audits and validation procedures cross-referenced extracted data with electronic records' source documents to ensure accuracy and protocol compliance.

Researchers conducted anthropometric measurements using approved bioelectrical impedance analysis machines and standard stadiometers, following PHCC clinical guidelines that meet international standards. BMI calculations and WC cutoffs followed World Health Organization recommendations [12]. Standard protocols were used by trained health professionals in the wellness centers to measure anthropometric indices. Two-time points were used for collection: pre-intervention as baseline and post-intervention at week 12.

Before starting the wellness center's physical exercise program, each patient had to undergo a standard PF assessment, including VO_2_ max and muscular endurance. The VO_2_ max was assessed using the 12-minute Cooper test when a patient runs or walks in precisely 12 minutes. This distance is then used to estimate VO_2_ max, using the calculation formula [13]:

$\text{VO}_2 \text{ max} = \frac{\text{distance in meters} - 504.9}{44.73}$

Muscle endurance was measured by how long a person could hold a plank or wall-sit position properly, and it was usually measured in seconds by a professional trainer. For push-ups, endurance is assessed by counting how many push-ups can be completed until fatigue [14].

Data analysis

Descriptive data, including numbers and percentages, were generated for each variable using crosstabulations. The research data were stored in a Microsoft Excel® file with a protective password accessible to the research team. The demographic and clinical characteristics of the patients were analyzed using descriptive statistics. Categorical variables, such as age and gender, were shown as percentages, while continuous variables were summarized using means and standard deviations. The mean differences in VO_2_ max and muscle endurance were calculated, and their statistical significance was assessed using the paired t-test. A box plot visually depicted changes in VO_2_ max and muscle endurance before and after the 12-week exercise program. We also explored how demographic characteristics and body measurements relate to changes in PF. IBM SPSS Statistics for Windows, Version 26 (Released 2019; IBM Corp., Armonk, NY, USA) was used for analyses, with a p-value of less than 0.05 indicating statistical significance.

## Results

Out of 909 participants, 739 were eligible, with an average age of 48.75 years ± 12.83 years. Approximately 74.56% of the patient data were from women, as detailed in Table 1. Before the intervention, the average body weight was 80.00 kg ± 14.71 kg. The average WC was 103.98 cm for men and 98.52 cm for women, with standard deviations of 12.51 cm and 13.04 cm, respectively. The pre-program BMI averaged 31.15 kg/m² ± 5.39 kg/m², and the average fat mass was 33.91 kg ± 8.42 kg.

**Table 1:** Demographic Characteristics of the Participants Before the 12-Week Physical Exercise (n = 739). SD: Standard Deviation; WC: Waist Circumference; BMI: Body Mass Index

Category	n (%)		Mean ± SD
Age Categories (Completed Years)			
18-30	70 (9.35%)	24.80 ± 3.24	
31-65	614 (81.98%)	49.49 ± 8.92	
>65	57 (8.68%)	70.98 ± 4.91	
Total	739	48.81 ± 8.30	
Gender			
Female	551 (74.56%)	-	
Male	188 (25.44%)	-	
Total	739	-	
Anthropometric Measurements			
Weight (Kg)	-	80.00 ± 14.71	
WC (cm)	Males	103.98 ± 12.51	
	Females	98.52 ± 13.04	
BMI (Kg/m^2^)	-	31.15 ± 5.39	
Fat Mass (Kg)	-	33.91 ± 8.42	

Table 2 illustrates pre- and post-intervention anthropometric measurements and PF parameters. After completing the program, the average changes in weight, WC for men and women, BMI, and fat mass were 0.95 kg, 0.99 cm, 2.34 cm, 0.27 kg/m², and 1.42 kg, respectively.

**Table 2:** Effect of 12-Week Physical Exercise on Physical Fitness Metrics and Anthropometric Measurements in Adults at Community Wellness Services, Primary Health Care Corporation. A p-value of less than 0.05 indicating statistical significance. WC: Waist Circumference; BMI: Body Mass Index; Pre-I: Pre-intervention; Post-I: Post-intervention; CI: Confidence Intervals; SD: Standard Deviation; Md: Mean Difference

Parameter	Md ± SD, Pre-intervention	Md ± SD, Post-intervention	Mean Difference	Paired t-test	p-value
Physical Fitness Metrics (VO₂ Max and Muscular Endurance)					
VO_2_ Max (mL/kg/min)	13.78 ± 6.66	16.25 ± 7.99	2.47	-9.13	<0.001
Push-Ups (No.)	16.69 ± 7.84	20.69 ± 7.83	4.00	-14.36	<0.001
L-Wall Set (sec.)	47.46 ± 38.56	65.48 ± 42.87	18.02	-12.97	<0.001
Plank (sec.)	42.17 ± 31.36	58.94 ± 35.52	16.77	-16.36	<0.001
Anthropometric Measurements					
Weight (Kg)	80.00 ± 14.71	79.05 ± 13.89	0.95	3.57	0.000
WC (cm) - Males	103.98 ± 12.51)	102.99 ± 12.22	0.99	2.60	0.010
WC (cm) - Females	98.52 ± 13.04	96.18 ± 13.12	2.34	8.78	0.000
BMI (Kg/m^2^)	31.15 ± 5.39	30.88 ± 5.09	0.27	3.07	0.000
Fat Mass (Kg)	33.91 ± 8.42	32.49 ± 7.35	1.42	8.73	0.000

As shown in Table 2, the PF metrics of VO_2_ max and muscular endurance, participants showed significant improvements in PF after the intervention. VO_2_ max increased from 13.78 ± 6.66 mL/kg/min before the intervention to 16.25 ± 7.99 mL/kg/min after the intervention, indicating enhanced aerobic capacity. Muscular endurance also improved, as demonstrated by an increase in the number of push-ups completed, from 16.69 ± 7.84 to 20.69 ± 7.83 repetitions. Furthermore, the wall sit test duration improved from 47.46 ± 38.56 seconds to 65.48 ± 43.87 seconds, and the plank hold duration increased from 42.17 ± 31.36 seconds to 58.94 ± 35.52 seconds.

**Figure 1:**
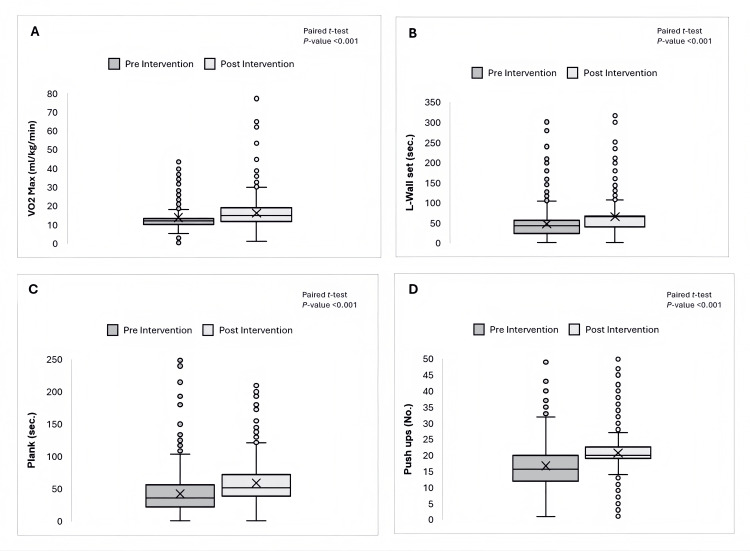
Effect of 12-Week Structured Physical Exercises Program on Fitness Metrics in Adults at Wellness Services, Primary Health Care Corporation. (A) Box plot showing the mean difference in VO_2_ max (mL/kg/min) between pre- and post-intervention. (B) Box plot showing the mean difference in L-Wall Sit (sec.) between pre-and post-intervention. (C) Box plot showing the mean difference in Plank Hold Time (sec.) between pre-and post-intervention. (D) Box plot showing the mean difference in Push-ups (No.) between pre-and post-intervention. All comparisons were analyzed using a paired t-test, with a p-value <0.001 indicating statistically significant improvements. A paired t-test demonstrated a statistically significant improvement in VO_2_ max, wall sit duration, plank duration, and the number of push-ups after the intervention (p < 0.05). Despite the presence of outliers, the observed improvements indicate a substantial impact of the intervention on physical fitness metrics. The statistical significance relation was determined using the paired t-test.

The paired t-test results for all parameters yielded p-values <0.001, confirming the effectiveness of the exercise intervention in improving overall PF, as illustrated in Figure 1.

In Table 3, the correlation matrix illustrates the relationships among PF metrics, age, gender, and anthropometric measurements using Pearson’s correlation testing. Age demonstrates a slight correlation with VO_2_ max (r = 0.061), suggesting that age has a limited direct effect on aerobic capacity. A minor negative correlation with push-ups (r = -0.056) indicates a slight decline in upper-body muscular endurance as age increases.

**Table 3:** Correlation Matrix Between Physical Fitness Metrics, Age, Gender, and Anthropometric Measurements. WC: Waist Circumference; BMI: Body Mass Index; Pearson’s correlation coefficient (r)

Variable	Age	Gender	Difference in VO_2_ max	Difference in Push-Ups	Difference in L-Wall Set	Difference in Plank	Difference in Weight	Difference in WC	Difference in BMI	Difference in Fat Mass
Age	1									
Gender	0.244	1								
Difference in VO_2_ max	0.061	0.067	1							
Difference in Push-Ups	-0.056	-0.085	-0.004	1						
Difference in L-Wall Set	0.044	-0.018	0.049	0.113	1					
Difference in Plank	0.040	0.051	0.199	0.130	0.179	1				
Difference in Weight	0.109	0.011	-0.090	0.019	-0.094	-0.094	1			
Difference in WC	0.054	0.045	-0.039	-0.054	-0.072	-0.074	0.352	1		
Difference in BMI	0.095	-0.008	-0.094	-0.024	-0.038	-0.097	0.681	0.486	1	
Difference in Fat Mass	0.015	0.019	-0.083	-0.009	-0.0138	-0.049	0.349	0.2532	0.368	1

Gender does not show strong correlations with PF metrics, suggesting that other factors, such as training history and lifestyle, may play a more significant role. VO_2_ max weakly correlates with body composition variables, exhibiting a slightly negative relationship with BMI (r = -0.094) and fat mass (r = -0.083), implying that higher body fat percentages may adversely affect aerobic fitness. Furthermore, push-ups display a weak negative correlation with gender (r = -0.085) and VO_2_ max (r = -0.004), indicating minimal associations between these factors. Other muscular endurance metrics, such as the plank and L-wall set, show weak correlations with anthropometric measurements, possibly due to individual variability in fitness levels.

## Discussion

This study evaluated VO_2_ max and muscular endurance changes among adults who participated in a 12-week structured physical exercise program within a wellness setting. The findings confirm the positive impact of structured physical exercise on enhancing aerobic capacity and muscular endurance. The significant increase in VO_2_ max after the intervention aligns with a previous study, which found that exercise training at various intensities effectively increases VO_2_ max in young, healthy adults, with 39 out of 40 study groups reporting positive effects [15]. Meta-regression analysis showed that the effect sizes were homogeneous, suggesting a consistent magnitude of VO_2_ max improvement across studies.

Furthermore, improvements in muscular endurance, evidenced by increased push-up repetitions, longer wall sit durations, and extended plank holds, underscore the importance of resistance and endurance training in boosting muscular strength and stability. Our findings align with previous research demonstrating that an eight-week exercise intervention in two consecutive years for older adults resulted in significant improvements in core strength endurance, with isometric trunk flexion and extension strength increasing by 21%-37% and moderate to large effect sizes. Notably, several fitness gains were maintained over a nine-month follow-up, highlighting the program’s effectiveness in sustaining physical improvements over time.

The observed decreases in weight, BMI, and WC indicate a favorable change in body composition, reinforcing the well-established connection between regular exercise and enhanced anthropometric measures. The correlation analysis revealed noteworthy associations between anthropometric variables and PF metrics. The moderate negative correlation between gender and fat percentage indicates that females in the study tended to have higher fat percentages than males. Additionally, the weak positive correlation between age and BMI suggests minor weight increases over time, while the weak negative correlation between age and fat percentage implies a slight reduction in fat mass with age. These findings align with previous studies exploring the interaction between body composition, age, and gender in response to structured physical activity interventions, indicating that physical activity influences body composition more significantly in men than in women, with men showing differences in 85.7% of parameters compared to 57.1% in women [16,17]. This disparity is largely attributed to higher circulating testosterone levels in men, which enhance variations in muscle and fat mass and, when combined with training, further amplify body composition changes in genders.

The statistical significance of the paired t-test results (p-values <0.001) for all PF parameters confirms the effectiveness of the 12-week exercise intervention in improving overall fitness. Regular participation in physical exercise programs provides extensive benefits that extend beyond immediate fitness gains. Improved cardiorespiratory fitness and muscular endurance significantly lessen the risk of cardiovascular diseases, obesity, and metabolic disorders, such as type 2 diabetes [18]. Engaging in structured physical activity aids in regulating blood pressure, enhancing insulin sensitivity, and supporting healthier lipid profiles. Moreover, maintaining an active lifestyle enhances mental well-being by reducing stress, anxiety, and depressive symptoms [19]. These long-term health benefits underscore the critical role of structured exercise programs in preventive healthcare strategies and public health policies.

The absence of a control group in this study poses a significant challenge in determining whether the improvements in PF are due to the physical exercise program or other factors, such as medication, lifestyle modifications, or dietary habits. This limitation hinders establishing a clear causal relationship between the intervention and the observed outcomes. To address this limitation, future research should employ randomized controlled trial designs, strengthening internal validity by controlling for confounders and thereby providing a more robust basis for establishing causality.

To sustain such long-term outcomes, researchers can draw from strategies like those discussed in studies on community-based physical activity programs. For instance, ensuring organizational support and integrating programs into existing healthcare infrastructure can improve sustainability. Additionally, objective measurement tools, such as wearable fitness trackers or accelerometers, can provide more accurate data on exercise intensity and adherence.

Moreover, this study relied on available data from the institutional medical record, with patient inclusion based on data availability rather than random recruitment. The overrepresentation of female participants reflects PHCC records, which indicate that women are the primary users of healthcare services in Qatar. This gender imbalance may limit the generalizability of the findings to males. Given known physiological differences in physical exercise between genders, future research should implement stratified sampling or balanced recruitment strategies to enable more comprehensive subgroup analyses.

Even with standardized measurement protocols and trained healthcare staff, there might still be some variability in how body measurements and PF were assessed, which could affect the results. The study also could not fully account for other lifestyle factors, like diet or exercise outside the wellness centers, which influenced the PF outcomes.

The study's advantage is its analysis of the institutional medical record data, which provides insights into the wellness program's effectiveness. Using actual community data was particularly valuable, as it captured the effectiveness of the physical exercise program on PF in a natural setting. Further investigation is needed to understand how different amounts of exercise affect PF. Even though the exercise program followed a set plan, differences in how intensely participants exercised and how well they stuck to the program affected the results. Future studies should use tools like fitness trackers to find the best exercise strategies and ensure that results can be repeated accurately.

## Conclusions

This study demonstrated the significant benefits of a 12-week structured physical exercise program in improving PF and body composition among adults in a wellness setting. The observed increases in aerobic capacity and muscular endurance, along with favorable changes in weight, BMI, and WC, reinforce the effectiveness of structured exercise in enhancing overall PF. The strong statistical significance of the results supports the incorporation of structured exercise programs into wellness initiatives to promote long-term health benefits and sustained physical improvements.
